# Malaria Vaccines: Progressing on a Bumpy Road

**DOI:** 10.1016/j.ebiom.2014.10.010

**Published:** 2014-10-23

**Authors:** Giampietro Corradin, Issa Nebie

**Affiliations:** a Biochemistry Department, University of Lausanne, 1066 Epalinges, Switzerland; b Centre National de Recherche et de Formation sur le Paludisme, Ouagadougou, Burkina Faso

Malaria remains the most prevalent parasitic infection in the world. Up to 40% of the world population is estimated to be at risk of contracting malaria. This figure may increase in the future with increasing temperature brought about by global warming. 207 million cases (uncertainty interval, 135–287 million) and 627,000 malaria deaths (uncertainty interval, 473,000–789,000) were estimated in 2013 (WHO, 2013). The majority of the deaths that result from *Plasmodium falciparum* infection (the most lethal of the *Plasmodium* species) occur among infants, children and prima–gravida women living in sub-Saharan Africa (WHO, 2013).

*Plasmodium vivax*, the second malaria species in terms of epidemiological importance but less deadly than *P. falciparum*, causes ~ 70–80 million clinical malaria cases annually with 2.85 billion people living at risk of infection (Guerra et al., 2010) and generates a substantial economic burden worldwide. This parasite species is prevalent mainly in Asia, Oceania, the Americas, and several countries of Africa, and accounts for half of the malaria cases outside Africa.

The current malaria-control tools in malaria-endemic countries rely on effective mosquito control and artemisinin-based case management. Challenges to controlling the burden of disease include the development of resistance of *Anopheles* mosquitoes to certain insecticides; the development of resistance of malaria parasites to chemotherapeutic agents (Baird, 2010); the absence of a gametocidal drug suitable for mass administration (White, 2004), and the risk of re-importation of malaria into geographic regions despite environmental elimination measures.

The development of a malaria vaccine is considered as one of the most cost-effective measures to counter the disease. Valuable progress has been achieved in the last 30 years in the development of *P. falciparum* subunit vaccines (Greenwood and Targett, 2011, Schwartz et al., 2012) that could be included in the Expanded Program of Immunization (EPI), whereas the *P. vivax* counterpart lags very much behind. Currently, the most advanced malaria vaccine candidate, the RTS,S vaccine based on the *P. falciparum* circumsporozoite (CS) protein, has gone through extensive testing in Africa where a recent phase 3 trial showed a 27 and 46% protection against clinical malaria in African infant and children respectively but unfortunately its efficacy wanes down in a relatively short time (The RTS, S Clinical Trials Partnership, 2014). The goal of the RTS,S Clinical Trial Partnership is now to seek a license for the first-generation malaria vaccine. Due to the fact that efficacy was different depending on the location and transmission rate, this intervention may be approved only for those regions where the efficacy was highest. In situations where the RTS,S efficacy is low, the limited available resources may be better used for other efficacious interventions such as artemisinin-based combination therapies (ACTs), insecticide-treated mosquito net (ITN) and indoor residual spraying (IRS).

In addition, prevision for a booster immunization may be considered, and clinical trials to determine its benefit must also be performed. This would further reduce resources needed for the interventions mentioned above. The homologation of the RTS,S vaccine may also pose a challenge to the development of alternative vaccines to increase the efficacy of RTS,S either as combination of RTS,S with new antigens or totally new formulations as discussed recently by Fowkes et al. (Fowkes et al., 2013), since any new formulation would need to be compared side-by-side with RTS,S vaccine. It is possible that the most effective combinations might derive from mixing vaccines that are found efficacious when tested alone, that target different stages of parasite life and/or are based on different mechanisms. In particular, one might speculate adding to RTS,S the RH5 antigen (whose antibodies are active in the in vitro inhibition of RBC infection by merozoites of heterologous *P. falciparum* strains), or antigens acting in the ADCI and/or opsonization as fragments from MSP2, MSP3, GLURP and PFF0165. Even though single antigens might be only weakly effective, their combination together with RTS,S is expected to be more effective in controlling the disease and, as a result, reducing transmission by mosquitoes (Carter and Chen, 1976).

To this end, efforts should also be made to develop a transmission-blocking vaccine (The MalERA Consultative Group on Vaccines, 2011). Known as “altruistic vaccination”, vaccinating in low-prevalence areas would have no direct benefit at the individual level but would protect their neighbors from becoming infected. This could be a stand-alone mosquito-stage vaccine or, more likely, a multi-component vaccine adding a mosquito-stage component to the partial transmission-blocking activity of a pre-erythrocytic vaccine. In parallel, more resources should be allocated for testing new *P. vivax* vaccines and to determine the mechanisms of protection for this species.

In most malaria-endemic countries, despite the large-scale distribution of ITN and ACTs, malaria still remains a serious public health problem. It is hoped that optimization of the current malaria interventions (e.g., ACTs, ITN, IRS) and the development of an effective and affordable malaria vaccine will bring an unprecedented rate of life saving and consequent improvement of life quality for the populations living in malaria-endemic countries.

The authors do not have any conflicts.
